# Acceptability of Adolescent COVID-19 Vaccination Among Adolescents and Parents of Adolescents — United States, April 15–23, 2021

**DOI:** 10.15585/mmwr.mm7028e1

**Published:** 2021-07-16

**Authors:** Aaron M. Scherer, Amber M. Gedlinske, Andrew M. Parker, Courtney A. Gidengil, Natoshia M. Askelson, Christine A. Petersen, Kate R. Woodworth, Megan C. Lindley

**Affiliations:** ^1^Department of Internal Medicine, University of Iowa, Iowa City; ^2^RAND Corporation, Santa Monica, California; ^3^Division of Infectious Diseases, Boston Children’s Hospital, Harvard Medical School, Boston, Massachusetts; ^4^Department of Community and Behavioral Health, University of Iowa, Iowa City; ^5^Department of Epidemiology, University of Iowa, Iowa City; ^6^CDC COVID-19 Response Team.

On May 10, 2021, the Food and Drug Administration (FDA) expanded its Emergency Use Authorization for the Pfizer-BioNTech COVID-19 vaccine to include adolescents aged 12–15 years; this authorization was followed by interim recommendations from the Advisory Committee on Immunization Practices (ACIP) for the vaccine among this age group (*1*). Using data from nonprobability–based Internet panel surveys administered by the Healthcare and Public Perceptions of Immunizations (HaPPI) Survey Collaborative, the acceptability of adolescent COVID-19 vaccination and self-reported factors increasing vaccination intent were assessed among independently recruited samples of 985 adolescents aged 13–17 years and 1,022 parents and guardians (parents) of adolescents aged 12–17 years during April 15–April 23, 2021, prior to vaccine authorization for this age group. Approximately one quarter (27.6%) of parents whose adolescents were already vaccine-eligible (i.e., aged 16–17 years) reported their adolescent had received ≥1 COVID-19 vaccine dose, similar to the proportion reported by vaccine-eligible adolescents aged 16–17 years (26.1%). However, vaccine receipt reported by parents of adolescents differed across demographic groups; parents identifying as female or Hispanic, or who had an education lower than a bachelor’s degree reported the lowest adolescent COVID-19 vaccination receipt. Among parents of unvaccinated adolescents aged 12–17 years, 55.5% reported they would “definitely” or “probably” have their adolescent receive a COVID-19 vaccination. Among unvaccinated adolescents aged 13–17 years, 51.7% reported they would “definitely” or “probably” receive a COVID-19 vaccination. Obtaining more information about adolescent COVID-19 vaccine safety and efficacy, as well as school COVID-19 vaccination requirements, were the most commonly reported factors that would increase vaccination intentions among both parents and adolescents. Federal, state, and local health officials and primary care professionals were the most trusted sources of COVID-19 vaccine information among both groups. Efforts focusing on clearly communicating to the public the benefits and safety of COVID-19 vaccination for adolescents, particularly by health care professionals, could help increase confidence in adolescent COVID-19 vaccine and vaccination coverage.

The HaPPI Survey Collaborative is part of a cooperative agreement between CDC and researchers at the University of Iowa and the RAND Corporation to survey health care professionals and the U.S. public on vaccine-related issues. Surveys to assess the acceptability of adolescent COVID-19 vaccination were administered during April 15–23, 2021, to U.S. adolescents aged 13–17 years and parents of U.S. adolescents aged 12–17 years using a nonprobability–based, independently recruited (i.e., nondyadic) Internet panel (Qualtrics, LLC).* Adolescents aged 13–17 years (rather than those aged 12–17 years) were surveyed because, under federal online privacy rules, after obtaining parental consent to join the panel, they can complete panel surveys without further parental consent (*2*). Sampling quotas by age, gender, and race and Hispanic ethnicity were used to reduce potential sampling bias.^†^ Among the 1,457 parents and 1,927 adolescents screened, 1,129 parents (77.5%) and 1,143 adolescents (59.3%) agreed to participate in the study. Among those agreeing to participate, 105 parents and 138 adolescents were excluded for providing low quality responses.^§^ The final samples included 1,022 parents (90.5% completion rate) and 985 adolescents (87.1% completion rate).

Among both adolescents and parents of adolescents, primary measures were self-reported COVID-19 vaccination (receipt of ≥1 dose) for adolescents aged 16–17 years and COVID-19 vaccination intentions for unvaccinated adolescents aged 12–17 years. Secondary measures were potential factors to increase vaccination intentions among respondents not reporting definite intent for adolescent vaccination, and trusted vaccination locations and information sources. Chi-square analyses were conducted to test for group differences using Stata (version 14.2; StataCorp, LLC).^¶^ To account for multiple comparisons, p-values ≤0.003 were considered statistically significant. This survey was reviewed and approved by the University of Iowa Institutional Review Board.**

Among parents with adolescents aged 16–17 years, 27.6% reported that their adolescent had received ≥1 COVID-19 vaccine dose (Table). Parent-reported adolescent receipt of a COVID-19 vaccine differed significantly when examined by parents’ gender, level of education, and race/ethnicity. Specifically, parents who identified as female, Hispanic, or who had less than a bachelor’s degree reported the lowest adolescent COVID-19 vaccination receipt. Among parents with unvaccinated adolescents aged 12–17 years, 55.5% reported that they “definitely will” or “probably will” have their adolescent child receive a COVID-19 vaccination. Parent-reported intent for their adolescent to receive a COVID-19 vaccination was significantly lower among female (49.3%) than among male (63.0%) parents and among those having less than a bachelor’s degree or living in the Midwest or South Census regions.^††^

**TABLE:** Reported adolescent COVID-19 vaccine uptake and intentions among U.S. parents and guardians (parents) of adolescents aged 12–17 years and adolescents aged 13–17 years, by respondent characteristics — United States, April 15–23, 2021

Respondent group/Characteristic	No.	% (95% CI)	COVID-19 vaccine receipt* for adolescents aged 16–17 years (95% CI)	Parental COVID-19 vaccination intentions^†^ for unvaccinated adolescents aged 12–17 years (or intentions^†^ of adolescents aged 13–17 years) (95% CI)
**Parents of adolescents aged 12–17 yrs**				
**Total**	1,022	100	27.6 (23.6–31.9)	55.5 (51.9–59.0)
**Adolescent age group, yrs** ^§^				
12**–**15	737	72.1 (69.3–74.8)	N/A	53.7 (46.8–60.5)
16**–**17	452	44.2 (41.2–47.9)	27.6 (23.6–31.9)	56.1 (52.0–60.2)
p-value	—	—	—	0.550
**Respondent gender**				
Male	524	51.3 (48.2–54.3)	35.2 (29.0–42.0)	63.0 (57.8–68.0)
Female	493	48.2 (45.2–51.4)	20.6 (15.9–26.2)	49.3 (44.5–54.1)
Transgender or other gender identity	5	0.5 (0.2–1.2)	—^¶^	—^¶^
p-value	—	—	0.001**	<0.001**
**Respondent race/ethnicity**				
White, non-Hispanic	650	63.6 (60.6–66.5)	33.0 (27.7–38.7)	57.5 (52.9–62.0)
Black, non-Hispanic	129	12.6 (10.8–14.8)	25.4 (15.9–38.1)	49.1 (39.6–58.6)
Hispanic	173	16.9 (14.7–19.3)	11.0 (5.8–19.9)	53.3 (45.3–61.1)
Other, non-Hispanic	70	6.9 (5.5–8.6)	26.7 (13.7–45.4)	57.4 (43.9–69.9)
p-value	—	—	0.001**	0.409
**Education** ^††^				
≤High school	181	17.7 (15.4–20.1)	22.1 (14.5–32.2)	40.5 (33.0–48.5)
Some college	258	25.2 (22.6–28.0)	16.9 (11.3–24.6)	47.4 (40.9–53.9)
≥Bachelor’s degree	583	57.1 (54.0–60.1)	35.0 (29.2–41.3)	66.2 (61.3–70.8)
p-value	—	—	0.001**	p<0.001**
**Urbanicity^§§^**				
Metropolitan	897	87.9 (85.8–89.8)	28.8 (24.5–33.4)	57.0 (53.2–60.7)
Micropolitan	65	6.4 (5.0–8.1)	21.9 (10.7–39.7)	38.5 (26.2–52.4)
Small town/Rural	58	5.7 (4.4–7.3)	16.7 (6.2–37.5)	53.1 (39.1–66.6)
p-value	—	—	0.329	0.033
**U.S. Census region**				
Northeast	205	20.1 (17.8–22.7)	37.9 (28.7–48.1)	66.4 (58.2–73.8)
Midwest	195	19.1(16.7–21.6)	26.3 (18.4–36.1)	46.9 (39.0–55.1)
South	400	39.2 (36.3–42.3)	26.3 (20.0–33.7)	50.8 (45.2–56.4)
West	221	21.7 (19.2–24.2)	21.0 (14.1–30.2)	62.4 (54.8–69.3)
p-value	—	—	0.059	0.001**
**Adolescents aged 13–17 yrs**				
**Total**	985	100	26.1 (22.7–29.8)	51.7 (48.3–55.1)
**Adolescent age group, yrs^§^**				
13–15	398	40.4 (37.4–43.5)	N/A	54.5 (49.6–59.4)
16–17	587	59.6 (56.5–62.6)	26.1 (22.7–29.8)	49.1 (44.4–53.8)
p-value	—	—	—	0.116
**Respondent gender**				
Male	358	36.4 (33.6–39.7)	23.7 (18.5–29.9)	49.5 (43.9–55.1)
Female	580	58.9 (55.6–61.8)	27.8 (23.3–32.7)	50.5 (46.1–55.0)
Transgender or other gender identity	47	4.8 (3.5–6.2)	—^¶^	—^¶^
p-value	—	—	0.286	0.783
**Respondent race/ethnicity**				
White, non-Hispanic	625	63.5 (60.4–66.5)	24.8 (20.4–29.9)	52.0 (47.8–56.2)
Black, non-Hispanic	130	13.2 (11.1–15.4)	19.8 (12.8–29.3)	42.9 (34.0–52.2)
Hispanic	180	18.3 (16.0–20.9)	31.2 (24.1–39.4)	51.5 (43.1–59.8)
Other, non-Hispanic	50	5.1 (3.9–6.8)	32.4 (19.3–49.1)	73.7 (57.4–85.4)
p-value	—	—	0.185	0.012
**Urbanicity^§§^**				
Metropolitan	838	86.9 (84.6–88.9)	26.9 (23.2–31.0)	53.3 (49.6–57.0)
Micropolitan	76	7.9 (6.4–9.8)	18.2 (9.3–32.6)	42.6 (31.4–54.7)
Small town/Rural	50	5.2 (4.0–6.8)	28.6 (14.8–48.0)	40.5 (26.7–55.9)
p-value	—	—	0.433	0.078
**U.S. Census region** ^¶¶^				
Northeast	188	19.2 (17.1–22.1)	24.8 (17.6–33.6)	61.3 (53.5–68.5)
Midwest	217	22.1 (19.4–24.7)	23.5 (16.7–32.0)	46.6 (39.5–53.7)
South	362	36.9 (34.1–40.2)	24.8 (19.6–30.8)	46.7 (41.2–52.4)
West	213	21.7 (19.0–24.2)	32.5 (24.9–41.2)	57.6 (50.0–64.8)
p-value	—	—	0.332	0.004

**Abbreviations:** CI = confidence interval; N/A = not applicable.

* Reporting receipt of ≥1 COVID-19 vaccine dose. Reported proportions might not sum to 100% because of rounding.

^†^ Percentage of respondents indicating the unvaccinated adolescent aged 12–17 years “definitely will get a vaccine” or “probably will get a vaccine.” This question was asked of 766 parents who reported having an unvaccinated adolescent aged 12–17 years and 832 adolescents aged 13–17 years who reported being unvaccinated. Reported proportions might not sum to 100% because of rounding.

^§^ Parents could indicate that they had adolescents in one or both age groups.

^¶^ Value had a relative standard error of ≥30%, indicating an unstable estimate that should not be reported. Statistical comparisons by gender include only respondents identifying as male or female.

** Indicates a significant group difference based on chi-square analysis with p≤0.003 (adjusted for multiple comparisons: 0.05/16 = 0.003).

^††^ Education level not applicable to adolescents.

^§§^ Categories created by collapsing across zip code–based Rural-Urban Commuting Area codes from the U.S. Department of Agriculture Economic Research Service.

^¶¶^ *Northeast*: Connecticut, Maine, Massachusetts, New Hampshire, New Jersey, New York, Pennsylvania, Rhode Island, and Vermont; *Midwest*: Illinois, Indiana, Iowa, Kansas, Michigan, Minnesota, Missouri, Nebraska, North Dakota, Ohio, South Dakota, and Wisconsin; *South*: Alabama, Arkansas, Delaware, District of Columbia, Florida, Georgia, Kentucky, Louisiana, Maryland, Mississippi, North Carolina, Oklahoma, South Carolina, Tennessee, Texas, Virginia, and West Virginia; *West*: Alaska, Arizona, California, Colorado, Hawaii, Idaho, Montana, Nevada, New Mexico, Oregon, Utah, Washington, and Wyoming.

Among adolescent respondents aged 16–17 years, 26.1% reported that they had received ≥1 COVID-19 vaccine dose. Among unvaccinated adolescents aged 13–17 years, 51.7% reported they “definitely will” or “probably will” receive a COVID-19 vaccination. Adolescent-reported COVID-19 vaccination receipt and intention to be vaccinated were similar across demographic groups.

Among 511 of 766 (66.7%) parents of unvaccinated adolescents who did not indicate that they “definitely will get the vaccine” for their adolescent, the most commonly reported factors that would increase intent for adolescent COVID-19 vaccination were having more information about safety (16.3%) and efficacy (13.4%) of COVID-19 vaccines for adolescents and having vaccination be a school requirement (13.2%) (Figure 1). Similarly, among 705 of 832 (84.7%) unvaccinated adolescents who did not indicate that they “definitely will get the vaccine,” the most commonly reported factors that would increase vaccination intent were more information about safety (21.7%) and efficacy (17.6%) of COVID-19 vaccines for adolescents and that vaccination be a school requirement (23.9%). Other potential factors that might increase vaccination selected by a relatively large proportion of adolescents included preventing the spread of COVID-19 to family and friends (17.1%), allowing resumption of or increase in social activities (15.5%), or traveling (14.5%). Very few parents (9.9%) or adolescents (8.9%) selected a COVID-19 vaccine recommendation by a health care professional as a factor that would increase vaccination intentions.

**FIGURE 1:**
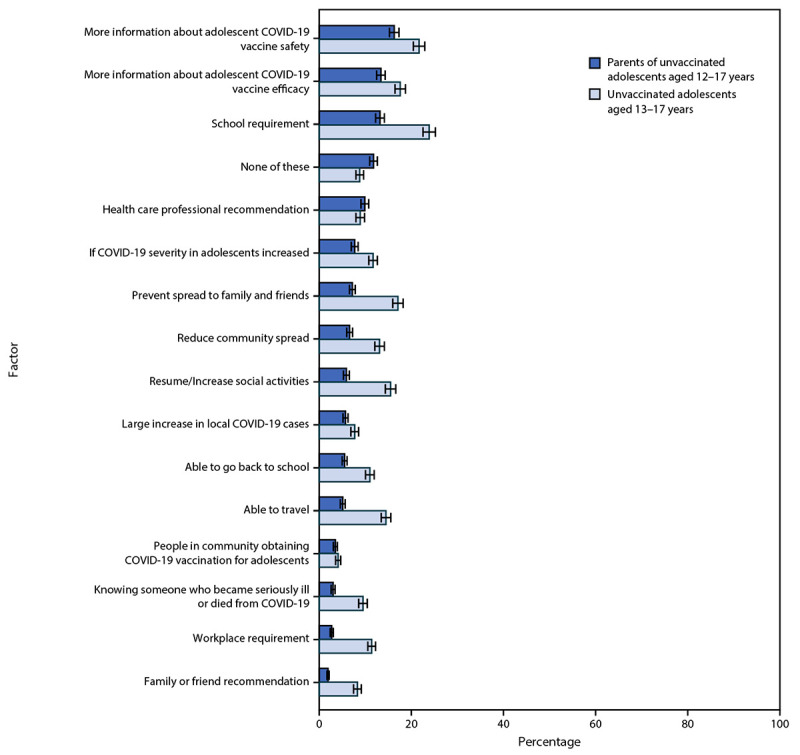
Factors that would increase adolescent COVID-19 vaccination intent according to U.S. parents and guardians (parents) of unvaccinated adolescents aged 12–17 years and unvaccinated adolescents aged 13–17 years who did not indicate definite intent to receive the vaccine* — United States, April 15–23, 2021 * Error bars indicate +/- 1 standard error.

Parents of unvaccinated adolescents and unvaccinated adolescents reported feeling most comfortable with vaccination occurring at the adolescent’s usual doctor’s office or clinic (66.6% and 76.5%, respectively). At least one quarter of parents of unvaccinated adolescents and one quarter of unvaccinated adolescents also reported being comfortable with vaccination at a local pharmacy (37.1% and 39.9%, respectively), a doctor’s office or clinic other than the usual one (32.2% and 31.8%), temporary indoor vaccination clinics (28.2% and 25.3%), or at school with a parent or caregiver present (26.1% and 30.2%). Approximately one half of parents of adolescents and one half of adolescents reported government agencies including CDC and FDA (53.1% and 57.8%, respectively), primary care professionals (47.3% and 45.7%), and state or local health officials (46.6% and 49.4%, respectively) as trusted sources of COVID-19 vaccine information (Figure 2).

**FIGURE 2:**
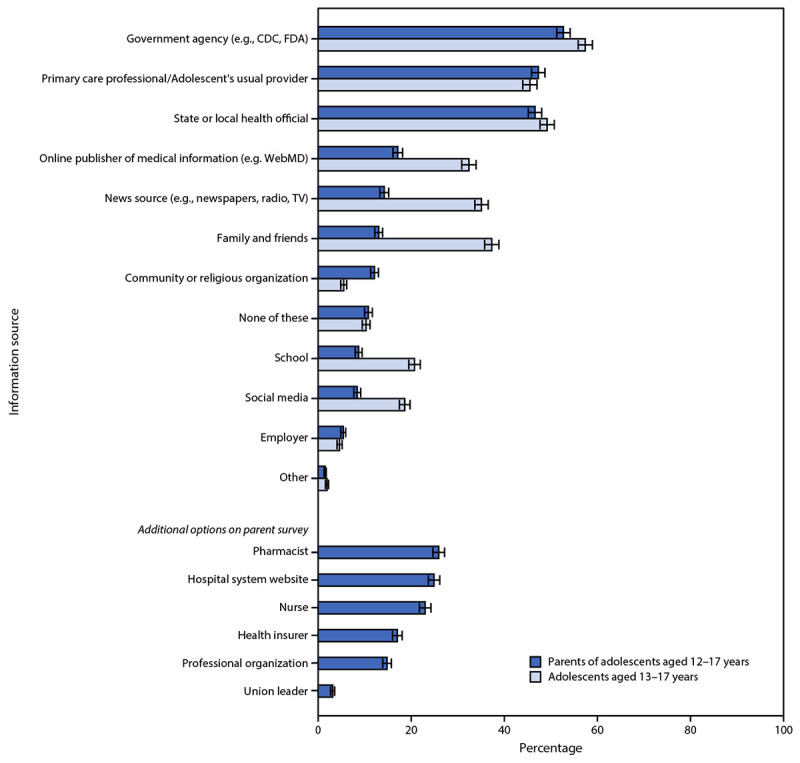
Trusted COVID-19 vaccine information sources according to U.S. parents and guardians (parents) of adolescents aged 12–17 years and adolescents aged 13–17 years* — United States, April 15–23, 2021 **Abbreviations**: FDA = Food and Drug Administration; TV = television. * Error bars indicate +/- 1 standard error.

## Discussion

Nonprobability–based surveys conducted just before the expanded availability of the Pfizer-BioNTech vaccine to adolescents aged 12–15 years found that approximately one half of unvaccinated adolescents and parents of unvaccinated adolescents reported not intending for or being uncertain about whether the adolescent would receive a COVID-19 vaccination. Female parents, those with lower educational attainment, and those living in the Midwest or South Census regions had lower intentions to have their unvaccinated adolescent receive a COVID-19 vaccination.

Most adolescents and parents reported being comfortable with adolescent COVID-19 vaccination occurring at the adolescent’s usual doctor’s office or clinic. However, other possible vaccination locations, such as local pharmacies or temporary indoor vaccination clinics, were also acceptable to many parents and adolescents. Public health officials at federal, state, and local levels and primary care professionals were the most trusted sources of information about COVID-19 vaccines. Having more information about adolescent COVID-19 vaccine safety and vaccine efficacy were among the most frequently selected factors that parents and adolescents reported would increase adolescent COVID-19 vaccination intent. Although few adolescents and parents of adolescents reported that a health care professional recommendation would increase their intent for adolescent COVID-19 vaccination, health care professionals were one of the most trusted sources of COVID-19 vaccine information. Given that a health care professional’s recommendation is one of the strongest predictors of vaccination in general (*3*), public health officials and primary care professionals can emphasize adolescent COVID-19 vaccine safety and efficacy in discussions with the public to help increase COVID-19 vaccination intent and coverage among adolescents.

The findings in this report are subject to at least three limitations. First, a nonprobability, quota-based sample was used, which increases the potential for bias and limits generalizability (*4*). For example, U.S. adolescents whose parents allow them to join Internet panels might be different from U.S. adolescents overall; however, the increased autonomy afforded by parents to these adolescents might also extend to medical decisions, potentially providing unique insights into factors affecting self-directed adolescent COVID-19 vaccination compared with parent-directed vaccination. Second, the surveys were administered online and only available in English, which could yield underrepresentation of U.S. residents without Internet access or those who have limited English proficiency. Finally, although statistical testing was conducted, small sample sizes in some demographic subgroups might limit the ability to provide estimates or compare findings, and the underlying sample might be subject to biases that cannot be quantified. These surveys were conducted before FDA authorization of COVID-19 vaccine for younger adolescents; parental and adolescent intent related to adolescent COVID-19 vaccination might differ now that vaccination is authorized.

As of July 6, 2021, approximately 8.3 million adolescents aged 12–17 years had received ≥1 dose of a COVID-19 vaccine (*5*). However, nearly one half of the unvaccinated adolescents and parents of unvaccinated adolescents in the current study indicated they were “unsure about,” “probably will not,” or “definitely will not” receive or have their adolescent child receive a COVID-19 vaccination. Given that having more information on adolescent COVID-19 vaccine safety and efficacy were primary factors identified by parents and adolescents as increasing vaccination intent, relaying this information to the public, particularly by public health officials and health care professionals, could help increase COVID-19 vaccine confidence, acceptance, and coverage among adolescents. In addition, efforts to enhance adolescent COVID-19 vaccination outreach to groups with lower reported adolescent COVID-19 vaccination intent might increase vaccine confidence and coverage among adolescents in the United States. Outreach and communication efforts should consider that adolescents might have different COVID-19-related risk perceptions, information needs, and messaging preferences than do adults (*6*). Efforts to increase vaccination coverage for adolescents will likely intensify prior to school reopening and could be adapted based on additional research to help determine how to communicate the benefits and safety of adolescent COVID-19 vaccination most effectively to adolescents and their parents.

**Summary**

**What is already known about this topic?**

Pfizer-BioNTech’s COVID-19 vaccine was authorized by the Food and Drug Administration and recommended by the Advisory Committee on Immunization Practices in May 2021 for adolescents aged 12–15 years.

**What is added by this report?**

In April 2021, 52% of unvaccinated adolescents aged 13–17 years and 56% of parents of unvaccinated adolescents aged 12–17 years reported intent for adolescent COVID-19 vaccination. The most common factors that would increase vaccination intent were receiving more information about adolescent COVID-19 vaccine safety and efficacy.

**What are the implications for public health practice?**

Efforts focusing on effectively communicating the benefits and safety of COVID-19 vaccination for adolescents to the public could help increase adolescent COVID-19 vaccine confidence and vaccination coverage.

## Footnotes

*
https://osf.io/nq94h/?view_only = ea1a744cc8a343b59bfa31f8ec1ae4fc

† Sampling quotas were used by age group, gender, and race and Hispanic ethnicity. For age group, the quota included 40% of adolescents aged 12–15 years (parent sample)/13–15 years (adolescent sample), 40% aged 16–17 years, and 20% not specified. Parents were able to report whether they had adolescents in both age groups. For gender, the quota included: 40% identifying as male, 40% identifying as female, and 20% not specified. For race and Hispanic ethnicity, the quota included 62.3% non-Hispanic White, 12.4% non-Hispanic Black, 17.3% Hispanic, and 8.0% Other race/ethnicity.

§ Low-quality responses included completing the survey too rapidly and exiting the survey before completion, and also included adolescents aged 12–15 years who indicated they had already received the COVID-19 vaccine.

¶ Analyses with weights to correct for potential biases in the distribution of adolescents and parents of adolescents across U.S. Census regions were also conducted. The unweighted and weighted analyses produced similar results. Therefore, unweighted results are reported for simplicity.

** 45 C.F.R. part 46; 21 C.F.R. part 56.

†† Midwest U.S. Census region: Illinois, Indiana, Iowa, Kansas, Michigan, Minnesota, Missouri, Nebraska, North Dakota, Ohio, South Dakota, and Wisconsin. South U.S. Census region: Alabama, Arkansas, Delaware, District of Columbia, Florida, Georgia, Kentucky, Louisiana, Maryland, Mississippi, North Carolina, Oklahoma, South Carolina, Tennessee, Texas, Virginia, and West Virginia.
